# Lectures on the Feeding of Infants

**Published:** 1905-01-14

**Authors:** James Burnet

**Affiliations:** Registrar Royal Hospital for Sick Children; Senior Clinical Tutor, Extramural Wards Royal Infirmary; Physician to The Marshall Street Dispensary, Edinburgh


### HOSPITAL CLINICS.

### LECTURES ON THE FEEDING OF INFANTS.

By JAMES BURNET, M.A., M.B., M.R.C.P., Edin. Registrar Royal Hospital for Sick Children; Senior Clinical Tutor, Extramural Wards Royal Infirmary; Physician to The Marshall Street Dispensary, Edinburgh.

### III.—ARTIFICIAL FOODS.

In this lecture we shall consider those preparations which are sold under the name of infants' foods, omitting all reference to the varieties of condensed milk which we have already touched on in a previous lecture. The number of artificial foods is legion, and accordingly we can only hope to make some general references to the subject at this time.

*Classification of Infants' Foods.*

When we come to analyse the various constituents of these foods we find that they readily admit of being roughly classified into three distinct groups:—

1. *Those containing Milk and Cereals.*—The best example of this variety is found in Horlick's malted milk. The starch contained in it is altered and it possesses a considerable amount of bicarbonate of soda. Another well-known example is Nestlé's milk food, which contains a very large proportion of unaltered carbohydrate in the form of starch.

2. *Those containing Cereals with the Starch* (a) *Altogether Unaltered,* (b) *Incompletely Altered.*—Ridge's and Neave's foods belong to the former subclass of this group. These both contain an extremely large amount of starch, probably about eighty per cent. Savory and Moore's food and the Allenbury malted food, while containing some unaltered starch, have a large percentage of it completely changed.

3. *Those containing Cereals with the Starch completely Changed.*—Of this class Mellin's food forms by far the best and most typical example. It contains nearly seventy per cent. of soluble carbohydrates, but not a trace of unaltered starch.

*When May Artificial Foods be Given?*

The answer to this question is very simple. They should only be given as a general rule after the child has cut some of its teeth. To this rule there is but one exception. They may be given temporarily at an earlier period when the digestion of ordinary milk mixtures causes difficulty. Thus we have frequently found the use of Mellin's food, or of Savory and Moore's, or of the Allenbury food advisable in cases where the milk when given with water alone was being constantly vomited. These foods, however, should rarely be employed as an expedient in treatment before the third or fourth month, and certainly never before all other means have been found unavailing.

*Why Are Artificial Foods Harmful?*

Most of them contain starch either completely or partially unconverted into soluble carbohydrates. If we reflect for a moment we will find that mother's milk contains no starch, and hence these so-called "foods" do not conform to the original composition of the infant's natural food. Starch can only be converted by means of the saliva and by the pancreatic secretion. These are not abundant or active until after the fifth or sixth month, and their functions of converting starch are not fully developed until after the first year of life. It is for this reason that those foods which contain starch are not at all suitable for young infants. If too much starchy food is given the child will become pale, even though it becomes fat and puts on weight. It will grow daily less vigorous, and will in time develop well-marked rachitis, usually of the anæmic type.

Another point, often overlooked, is that the composition of the food as advertised by the manufacturers becomes manifestly altered when the food is prepared according to the special directions given. Frequently the analysis indicates that the food contains exactly what the infant requires; but were we to analyse it when actually prepared we would often be astonished at the difference in the figures. A common habit of medical men is to have the names of half-a-dozen artificial foods in their memory, and when one fails to give satisfactory results, another and still another is tried until the list is exhausted. None is given a chance, and so all are straightway condemned. We have often found an infant vomiting a proprietary food who began to thrive at once on a simple milk and barley-water mixture. Mothers, and we fear even medical men in some cases, are impressed by the name "food," which is applied to all these artificial substances; but in many instances the term "food" is quite a misnomer.

*The Value of Artificial Foods is Displayed after the Period of Weaning.*

It is after the infant has been weaned, when we wish to add to the dietary, that these artificial foods come into use. At this time Mellin's food will be found a useful adjunct. It contains, however, very little fat, and therefore can never take the place of such things as porridge and eggs. Savory and Moore's food is also good, and we can recommend the well-known Allenbury food No. 3, at this period. Milo food, recently introduced by the Nestlé Company, is likewise a valuable one. Hovis food No. 2, we may specially mention, as it seems readily digested, and children are very fond of it. These foods are probably the most reliable of all, and one or other of them, prepared according to the makers' directions, will serve as a useful variety in the infant's dietary.

*Diet after Weaning.*

In addition to giving artificial foods, milk should still form the principal basis of the diet. Cream should be added to this; and half an egg beaten up with bread crumb should be given every other day. Towards the end of the first year a little well-boiled porridge may be taken with advantage. In order to guard against the possibility of scorbutus in warm weather, when all milk ought to be boiled, a little orange or grape-juice should be given daily.

*Diet during the Second Year.*

This is the time when the child very frequently begins to sit at the table with its parent, and it is then apt to be given tea, soup, beef, potatoes, fish, and other articles quite indiscriminately, to the almost total exclusion of milk. Now, of course, this is most injurious. Another common mistake made by medical men in such cases is to order a completely farinaceous diet. This usually serves to aggravate matters by producing abdominal distension, flatulence, and an increase of the rachitic symptoms which we desire to avoid. The following is a suggested table of diet suitable for use during the second year :—

7 A.M.—A cupful of boiled milk.
9 A.M.—Well-boiled porridge and milk; a thin slice of bread and butter.
12.30 P.M.—Well-mashed potato and gravy; softly-boiled egg, with breadcrumb; a few tablespoonfu's of custard pudding.
4.30 P.M.—A cupful of Mellin's or other food made with milk; a thin slice of bread and butter.
8 P.M.—A cupful of boiled milk, with rusk.

Care should be taken that the child is not fed between meals, and that no sweets are given, or pastries of any kind. He should have a certain amount of plain water every day, especially in the forenoon and afternoon. This often helps materially in keeping the bowels regular in action. Towards the end of the second year the child should have mince, fish, pounded beef or chicken, and clear soups, while well-baked apples are allowable.

*Results of Improper Feeding.*

The too early and continued use of artificial foods leads to rachitis and scorbutus, with their attendant phenomena. The same result is brought about by giving articles of food, such as tea, biscuits, fish, etc., which are not suited to the age of the child. Artificial foods are also liable to cause diarrhœa and vomiting. The first evidences, however, that the infant is not thriving will be shown in its general condition. It will become peevish and restless, rolling its head about on the pillow. During sleep the scalp will become quite moist. The abdomen will be unduly prominent, and the fontanelle long in closing. There will be a tendency to nasal and bronchial catarrh. Should the nervous system become affected, crowing and fits may take place, while tetany may even supervene. The infant will be slow in cutting his teeth, and will be late in walking. He will probably become fat, but at the same time will be exceedingly soft and flabby; and he will scream whenever he is handled or lifted out of his crib. Such infants often become extraordinarily pale, with enlargement both of liver and spleen. The cause of these phenomena lies in the want of fat in the food. The rapidly-growing organism of the infant evidently requires an enormous amount of fat for its maintainence, and hence when this is insufficiently supplied in the food the infant's development does not follow the healthy standard.

*How can we Supply the Deficiency in Fat?*

We may do so first by attending to the diet. Oatmeal, eggs, cream, and butter contain a large percentage of fat, and accordingly these should be given largely to such infants. The oatmeal used should be fine, and it should be well boiled and given with milk or cream. Eggs may be given softly boiled and beaten up with breadcrumb, or they may be poached or even scrambled. Some children cannot be made to take eggs, and in such cases they may be given in the form of a custard. Bread and butter, or even dripping obtained from roast beef, should be given at breakfast and also at the afternoon meal. Another mode of supplying the defective fat is to administer cod-liver oil, either plain or in the form of an emulsion. The latter is best given as Nutrol.[1] Lately I have been trying Virol, and find it an excellent substitute for cod-liver oil. It consists of eggs, bone-marrow, and malt, and is delightfully flavoured. From its composition it would appear to contain a large percentage of fat, and that it does so is proved by the good results obtained from its administration to rachitic and anæmic babies. It is best given plain, but it may be mixed with the food if preferred. Another advantage which it possesses is that it can be given during the hot summer months, when cod-liver oil usually causes considerable gastro-intestinal disturbance. It certainly should be employed instead of cod-liver oil whenever the latter is found to disagree, if, indeed, it is not given altogether as a more satisfactory substitute.

In conclusion I would counsel the practitioner against advising artificial foods instead of ordering milk mixtures, which are undoubtedly most suited to the average infant's needs. Before prescribing any food he should further satisfy himself as to its exact composition, remembering that those containing unaltered starch are certain to induce rachitis and a condition of general malnutrition. Again many artificial so-called "infants' foods" show an analysis which fairly represents the infant's requirements; but it is well to bear in mind that many of these foods, when diluted and mixed according to the given directions, fall very far short in regard to their percentage composition. Medical men at the present time apparently fail to recognise the irreparable harm that may be done by the indiscriminate use of infants' foods, and it is only by their careful attention to the general principles which underlie infant feeding that a change in the present evil tendency can be expected to take place. If these brief lectures help in some small measure to remedy the existing need for proper infant feeding, my object in writing them will have been fully accomplished.

[1] A preparation made for me by Mr. Davidson, of Edinburgh, and containing 70 per cent. of genuine cod-liver oil.

